# Protocol for Nuestro Sueño: A randomized trial of a couples-based intervention to improve PAP adherence and sleep health among Hispanic patients beginning positive airway pressure (PAP) and their partners

**DOI:** 10.1016/j.sleep.2026.109019

**Published:** 2026-05-15

**Authors:** Kelly Glazer Baron, Carmela Alcántara, Ivette López, Matthew Euler, Brian R.W. Baucom, Bobbie Bermudez, Yeny Arones, Sara Carbajal-Salisbury, Giannyn Fabian, Michael Grandner, Suzanne Gorovoy, Silvia Lopez, Sairam Parthasarathy, Wendy Troxel

**Affiliations:** a Department of Family and Preventive Medicine, University of Utah, USA; b School of Social Work, Columbia University, New York, NY, USA; c Department of Psychology, University of Utah, USA; d Eccles School of Medicine, University of Utah, USA; e Alliance Community Services, USA; f Mariposa Health, Tucson, AZ, USA; g Department of Psychiatry, University of Arizona, Tucson, AZ, USA; h Department of Medicine, University of Arizona, Tucson, AZ, USA; i Rand Corporation, Health Division, Pittsburgh, PA, USA

**Keywords:** Obstructive sleep apnea (OSA), Couple, Positive airway pressure (PAP), Adherence, Sleep, Transdiagnostic, BBTI, Hispanic, Treatment, Alzheimer's disease

## Abstract

**Background:**

Obstructive sleep apnea (OSA) is more common among Hispanic individuals and contributes to risk for cardiometabolic diseases, dementia and poor quality of life. Positive airway pressure (PAP) improves sleep and quality of life, and culturally adapted interventions are promising for increasing treatment engagement. The goal of this study is to test Nuestro Sueño, a culturally adapted couples-based intervention to promote positive airway pressure adherence and sleep health for Hispanic couples in which one partner is recently diagnosed with OSA.

**Methods:**

We are conducting a two-arm, parallel group, single blind, randomized controlled pilot/feasibility trial to compare our novel culturally adapted treatment to an information control (IC). Nuestro Sueño is a culturally adapted dyadic behavioral intervention based on a transdiagnostic model. The digital health program involves 3-weekly sessions delivered via telehealth with a community health worker. Content is focused on education about OSA and PAP, improving both partner's sleep quality, increasing partner support and communication, and couple-level goal-setting around sleep and PAP use. The IC includes standardized patient educational materials. Both groups receive the usual follow-up care. Assessments will be completed pre-treatment, 1 and 3 months after starting PAP. Our main outcomes are feasibility and treatment satisfaction. Secondary outcomes include comparing Nuestro Sueño to IC for PAP adherence, sleep quality (self-report and objective) and cognitive measures of memory, processing speed and verbal fluency.

**Discussion:**

Nuestro Sueño is a novel culturally adapted intervention focused on improving PAP adherence and sleep health among couples in which one partner is recently diagnosed with OSA. Results of this study will be used to inform the design of a subsequent fully adequately-powered clinical trial. If successful, this intervention could significantly advance current clinical practice in the treatment of OSA and sleep health more comprehensively as well as promote sleep health equity among Hispanic patients, who are more likely to face challenges to obtaining diagnosis and treatment for OSA.

**Trial registration:**

Clinicaltrials.gov, NCT06649929.

## 1. Background

Obstructive sleep apnea (OSA) is a prevalent, under-diagnosed, and under-treated sleep disorder among Hispanics [1]. OSA causes daytime sleepiness, decreased quality of life, increases risk for cardiometabolic disease and dementias [2–6]. The first-line treatment for OSA, positive airway pressure (PAP), is highly effective at treating OSA symptoms in the patient and improving sleep in both the patient and bedpartner, however approximately half of patients are non-adherent [7–9]. Despite higher rates of OSA risk factors among Latinos (e.g., obesity), they remain under-represented in OSA research [10]. There are significant racial and socioeconomic disparities in PAP adherence [11–13], though most of this work has focused on racial (i.e., Black versus White) differences. The limited available evidence about PAP adherence among Latino adults is mixed [14–16], suggesting considerable heterogeneity in PAP adherence based on national origin or sociocultural background, acculturation, and individual and neighborhood-level socioeconomic characteristics. For example, one study focused on Latino patients with OSA found that the patienťs perceived benefit with PAP therapy was associated with greater PAP adherence, whereas primary language was not associated with PAP adherence [14]. Another study found lower continuous positive airway pressure (CPAP) adherence in neighborhoods with greater proportions of Black or Latino residents [11].

There is a strong rationale for addressing PAP adherence using culturally adapted interventions, particularly within the context of the couple and family [17]. The consequences of OSA extend beyond the individual with OSA, also affecting the romantic partner or spouse [18]. For example, bedpartners of individuals with OSA are often “casualties” of untreated or poorly treated OSA [19], [20,21], as the nocturnal symptoms of OSA, including snoring, gasping for air, or choking are highly disruptive to the bedpartner's sleep and can contribute to hyper-vigilance, which is a risk factor for insomnia [22]. Indeed, partners of snorers are at 3 times greater risk of insomnia [23] and are also at elevated risk of depression and reduced quality of life (QOL) [20,21,24]. Family relationships are likely to be even more important among Hispanic patients. As represented by the core cultural value of *familismo*, interdependence among family members and the active maintenance of harmonious pro-social family relationships configures everyday social practices and decision making, such as with whom and where an individual lives and the role of family in important life decisions [17,25]. For example, Latino people are more likely to rely on family members to provide support and care, rather than non-familial supportive services [26] a key hypothesized protective factor for health among Latinos [27]. Existing PAP adherence interventions have been targeted exclusively towards the OSA patient, neglecting to consider the immediate interpersonal and broader sociocultural context [15], which is particularly important for racial and ethnic minority patients affected by health disparities. Given that reimbursement of PAP often depends on 90-day usage, such findings highlight the importance of considering broader sociocultural determinants of adherence and providing targeted and culturally adapted interventions to promote PAP adherence among minoritized populations in order to promote health equity.

Study team members Baron and Troxel developed and tested WePAP [28], a couples-based intervention for PAP adherence. WePAP was acceptable, feasible and showed promising effects on patient adherence, and both partners’ sleep quality as well as relationship quality. A limitation of our pilot was that our sample was primarily comprised of non-Hispanic White, highly educated patients (84% white, 43% held graduate degrees), and there was limited variability across conditions, as patients in both the intervention and information-control group had very high PAP adherence (>80% using PAP for more than 4 h per night at 3-month follow-up), though those in the WePAP group had higher non-statistically significant higher adherence than those in the information control group. As a next step, our team developed Nuestro Sueño, by adapting WePAP for Latino couples, utilizing the cultural adaptation framework by Barrera et al. [29] A cultural adaptation involves the systematic Utilizing both “top down” and “bottom up” approaches, our team utilized discussions with a community advisory board (CAB), focus groups and dyadic interviews to adapt language, content, visual images to create the 3 session Nuestro Sueño intervention.

A key adaptation of our intervention is that Nuestro Sueno will use Hispanic and bilingual (Spanish and English) community health workers (CHWs) to deliver our intervention to maximize treatment acceptability and most effectively reach Latino couples. CHWs are front-line public health workers who are trusted members of their communities and serve as liaisons between health/social services and their communities [30]. CHWs are integral to our intervention strategy because of their ability to deliver the intervention to ethnicity-, language- and culture-matched patients. CHWs have been used rarely in sleep interventions (two previous studies) [31,32], but research has supported their effectiveness in delivering interventions for diabetes [33] and hypertension management [34]. The in-depth cultural adaptation process is described in detail elsewhere [35]. The goal of this paper is to describe the study protocol for a pilot randomized controlled trial comparing the efficacy of Nuestro Sueño to an Information Control (IC) on PAP adherence. This intervention fills a critical gap in focusing on PAP adherence in an understudied community.

## 2. Design and methods

### 2.1. Study design overview

**Fig. 1.**
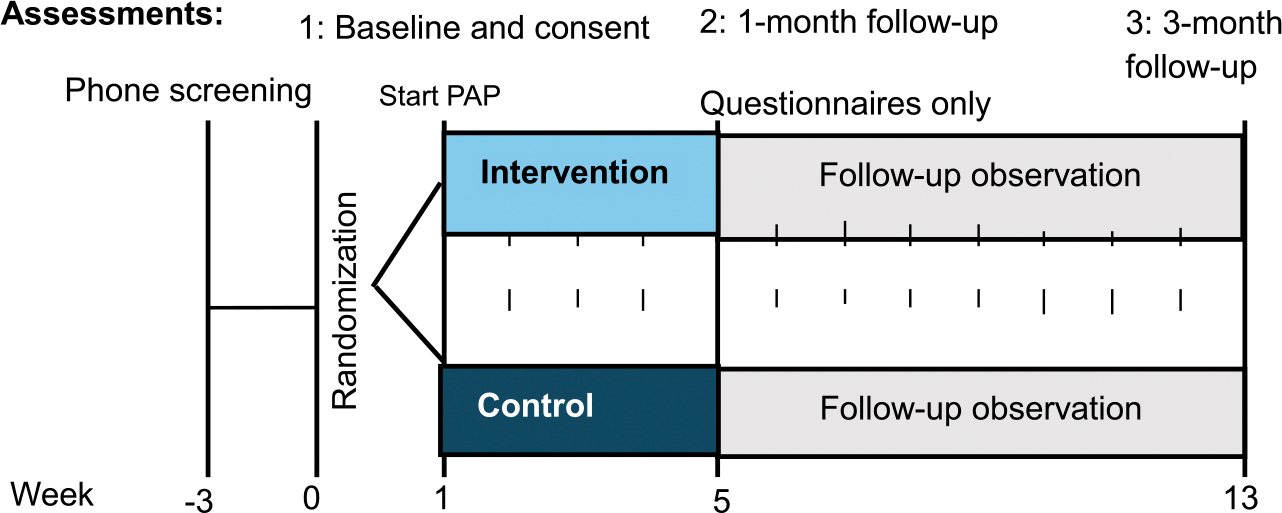
Study procedure.

This is a two-arm, parallel-group, single-blind, randomized controlled pilot trial to evaluate the feasibility and preliminary efficacy of “Nuestro Sueno”, couples-based intervention for Latino patients with OSA and their partners compared to an information control. Participants include Hispanic patients who are newly diagnosed with OSA and starting PAP therapy and their partners (*n* = 160, 80 couples). Couples will be recruited from two sites, the University of Utah Sleep Centers and the University of Arizona/Banner University Medical Center – Tucson (UA/BUMC-T) Sleep Clinics. The procedure is depicted in Fig. 1. After completing a pre-treatment baseline assessment, couples are randomly assigned to either a 3-session virtual sleep health intervention (We-PAP) or to a patient-focused information control group (IC) arm. Patients and partners complete follow-up assessments at post-treatment (approximately 1 month after starting PAP) and 3 months after starting PAP treatment. Approval for this on-going study was provided by the University of Utah Institutional Review Board (IRB_00177835) with a single site IRB with University of Arizona relying on the University of Utah approval. The study is registered with ClinicalTrials.gov (NCT06649929, 3/20/2025).

### 2.2. Community engagement framework

Our framework is based on the Participatory Research principles which are centered on creating true partnerships that are based on inclusive collaborations and power-sharing [36]. Utilizing concepts from the participatory research framework [37,38], the research plan includes collaboration from ethnicity-matched community members throughout the study. The team established the following principles and procedures: 1) The team approached the project with a value of mutual learning and trust between academic researchers and community partners (e.g., CHWs, CAB members). 2) Community advisory board was established in the first phase of the study, including 3 CHWs, 3 patients, 3 partners, 1 sleep medicine physician and the research team, 3) CHWs participate in the study as study team members and were included in all aspects of the study, including the process of developing the funding application, adaptation of the intervention and in ongoing study operations meetings, 4) CAB reviews, provides feedback, and approves of the intervention and study methods, including study advertising and surveys, 6) Participants and the CAB are provided with regular and timely feedback, and 7) The study team utilizes experiences in the study to promote education and increase awareness and treatment for sleep apnea in the communities participating in the study (e.g., creating and distributing information packets, screening questionnaires), such as attending health fairs and providing community presentations outside of work hours.

### 2.3. Theoretical framework and cultural adaptation process

Consistent with the WePAP program from which this treatment was adapted, Nuestro Sueño is based on the transdiagnostic sleep and circadian model (i.e., a treatment that addresses common components of multiple disorders rather than a single disorder) [39]. Consistent with key, evidence-based principles from couples-based interventions for other chronic health conditions, Nuestro Sueño conceptualizes PAP adherence and both partners' sleep health as a “shared” experience and delivers education and intervention through the lens of the couples’ shared sleep goals and challenges. The treatment provides education about the importance of sleep for health, then uses specific techniques to improve PAP adherence and insomnia symptoms based on best practices in cognitive behavioral (CBT) strategies [40–42].

We used a rigorous cultural adaptation framework [29] that progressed over four-stages to guide our process [35]. In stages one to three, 6 focus groups with 15 couples, and 6 dyadic interviews with members of the target population were conducted to obtain feedback. In stage four, a pilot study was conducted with four couples who were members of the population of interest. In total, 81 cultural adaptations were made to WePAP. Focus group discussions emphasized the importance of family in sleep, and the lack of information and misinformation about OSA in their countries of origin, as well as high interest in OSA education and treatment. As such, most adaptations were deep-level adaptations to the intervention content such as the integration of cultural values such as *familismo* and gender roles [i.e., marianismo] into treatment, and tailoring the content to be accessible across health literacy levels. Surface-level adaptations to WePAP included observable changes such as translation to culturally relevant language (Spanish), use of community health workers as interventionists, and integration of culturally relevant images and health information.

### 2.4. Recruitment, screening and consent

A total of 80 couples will be recruited from the University of Utah Health Sleep Centers and UA/BUMC-T. Patients who are undergoing testing (in-lab or home sleep testing) receive a letter introducing the study and notifying them they may be contacted about the study. At the University of Utah, study staff contact potential patients via phone and email to provide study information and if interested, conduct screening for study eligibility. At UA/BUMC-T, study staff review charts for upcoming patients and contact the sleep physician for permission to contact the patient. After the physician confirms the patient agrees to be contacted, study staff contact the patient. Interested couples schedule a virtual screening and consent visit over zoom to review and sign the online consent form and arrange to complete the baseline assessment. All study procedures are conducted in the couple's preferred language (either English or Spanish) by fluent bilingual and bicultural research staff.

### 2.5. Eligibility criteria

Inclusion criteria for the couple include: 1) Age ≥18 years; 2) Married or living with a romantic partner for at least 1 year; 3) Patient is PAP naïve or re-initiating PAP after 3 or more months; 4) Able to read and write in English or Spanish; 5) Able to access online, video-conference capabilities (Wi-Fi or cellular plan) on their own devices or a device provided by our study. Exclusion criteria include: 1) Self-reported diagnosis of severe comorbid sleep disorders other than insomnia (e.g., moderate or severe RLS; REM Behavior Disorder, narcolepsy); 2) Presence of severe medical and psychiatric disorders that would interfere with participation in treatment (schizophrenia, bipolar disorder, hemodialysis); 3) Patient is using supplemental oxygen or adaptive servo-ventilation, 4) overnight work >1 night per month.

### 2.6. Randomization and blinding

Upon completion of the baseline assessment, couples are randomly assigned to either Nuestro Sueno or IC by the study statistician (BB) using a randomization table developed using a random number generator. The randomization table has a 1:1 ratio of Nuestro Sueno to IC using permuted blocks of 4, 6, or 8, with equal strata for male and female patients. The randomization assignment is provided to the unblinded staff member (BB), who then contacts the CHW interventionist and couple via phone and email to notify them of their group assignment. Blinded team members include KB (PI overseeing recruitment and data collection), SV and ME, outcomes assessor and cognitive assessment supervisors and IL, who oversees the community engagement processes. Unblinded team members included CA and WT, who provide intervention supervision, statistician, and BB, research manager, who delivers the intervention assignment and the interventionists (YA, SC and GF).

### 2.7. Assessment schedule (Table 1)

At baseline/pretreatment, couples complete questionnaires, 7 days of Fitbit sleep monitoring and daily sleep diaries (delivered either via text message or on paper), and a brief cognitive testing battery. Approximately 1 month after beginning PAP and upon completion of their intervention, couples complete a second set of questionnaires. Finally, 3 months after starting PAP couples complete questionnaires, 7 days of sleep monitoring and daily sleep diaries and a brief cognitive testing battery. PAP use data are collected via download using the corresponding cloud-based systemTable 1.

### 2.8. Follow-up rationale

The primary adherence outcome of 3 months was selected based on the Medicare criteria Medicare criteria for % of nights ≥4 h, selecting the best 30 day window recorded within the first 90 days [43]. The 1 month follow-up was selected to assess earlier changes after starting PAP.

### 2.9. Intervention description (Table 2)

Nuestro Sueño was developed through an iterative process that included focus groups and a brief field trial before beginning the RCT. Couples assigned to the Nuestro Sueño intervention complete three online Zoom-hosted, videoconference-based intervention sessions conducted weekly, which utilize structured PowerPoint slides. All sessions include the themes of sleep education, dyadic coping and communication.

- **Session 1** (60 min) focuses on a program introduction, education about OSA, PAP and strategies for successfully beginning PAP and communication about PAP.
- **Session 2** (60 min) focuses on troubleshooting any issues with PAP, sleep health and strategies to improve poor sleep (techniques based in Brief Behavioral Treatment of Insomnia; BBTI)
- **Session 3** (60 min) continues the discussion about PAP troubleshooting and strategies for improving sleep health and introduces relaxation strategies (for adjusting to PAP and also for improving sleep health in general).

During each session, the interventionist reviews between session assignments (if applicable), presents session content, engages the couple in discussion and planning and assigns homework for the next session (if applicable). The educational components include the definition of obstructive sleep apnea, contributing factors and consequences for the patient and their family. Education about CPAP includes explanation about how CPAP reduces apnea, recommendations for use and techniques for overcoming initial challenges with the treatment. BBTI is an empirically validated treatment for chronic insomnia that was designed to be delivered by non-mental health interventionists [42]. The treatment focuses on education about the two processes that control sleep (homeostatic and circadian), sleep hygiene education, description of sleep restriction and stimulus control. BBTI techniques are reviewed for all couples, and for participants experiencing insomnia symptoms, the interventionist recommends changes to their sleep schedules according to the treatment manual.Table 2.

### 2.10. Information control (IC)

In this group, couples receive a packet of standardized patient educational materials in English and/or Spanish about OSA and PAP published by the Myapnea.org. In addition to notifying participants in this group of their group assignment, the interventionist contacts couples to ensure they have received the study materials. If the couple has questions about PAP, the interventionist refers couples to direct questions to their sleep medicine provider or durable medical equipment (DME) company.

### 2.11. Interventionist training, supervision, and fidelity monitoring

Nuestro Sueño is delivered by CHWs who completed standardized training and ongoing supervision throughout the trial. The CHWs attended 2 h of didactic training on OSA and CPAP treatment as well as a hands-on session with a sleep technologist at the sleep center (either University of Utah or University of Arizona) where they had the opportunity to discuss the process of CPAP set up and troubleshooting. Following the general training, the CHWs had 3, 90 min sessions where the study PIs (clinical psychologists with training in behavioral sleep medicine) reviewed each of the intervention sessions slide by slide and then the CHWs recorded role-plays of each session. The CHWs then were assigned two practice couples (usually a patient and partner who were established PAP users) and recorded 3 sessions. The practice couples were conducted in English and Spanish. After supervisor approval to deliver the intervention (i.e., >80% fidelity to intervention content), each CHW attends weekly supervision meetings with one of the study PIs to review recordings and discuss ongoing issues. CHWs rate each session for fidelity via a structured checklist completed at the end of each session. In addition, a random sample of 10% of sessions will be rated for fidelity by one of the lead investigators.

### 2.12. Measures

Measures are administered in English and Spanish, using validated translations when available. For measures that were not available in Spanish, they were translated by native a Spanish speaker and then back-translated.

#### 2.12.1. Feasibility and acceptability measures - primary outcomes

We will use a combination of quantitative and qualitative measures to assess the feasibility and acceptability of the study. Such methods include percentage of participants completing intervention sessions, ratings of ease of participation in the telehealth, questions about the number and duration of sessions, and perceived benefits of the intervention and enjoyment of the sessions. Additionally, couples will be asked open-ended questions about the format, feedback on the content, materials and general feedback and suggestions for improving the interventions.

#### 2.12.2. Secondary outcome measures

##### 2.12.2.1. PAP adherence.

Patient PAP adherence is recorded by the patienťs PAP machine and remotely downloaded. PAP adherence is measured continuously. The main time point for adherence is at 3 months and the main adherence measure will be % of nights with use ≥4 h. We will also analyze the % of nights used, average duration used per night and nights skipped. In sensitivity analyses, we will evaluate the Medicare criteria for % of nights ≥4 h, selecting the best 30 day window recorded within the first 90 days [43].

##### 2.12.2.2. Objective sleep measures.

Sleep is estimated using Fitbit Inspire 3 devices. Sleep data are automatically recorded and downloaded using the open application programming interface (API). Sleep data are considered valid if at least 3 h of sleep are recorded. When multiple sleep periods are detected (such as situations with high wake after sleep onset), sleep periods within 2 h are considered part of the same sleep interval. Sleep variables recorded include sleep duration and sleep efficiency. The primary objective sleep measure will be efficiency, calculated as total sleep time within the automatically recorded sleep interval.

##### 2.12.2.3. Sleep diary.

Participants complete a modified Consensus Sleep Diary [44] to quantitatively assess dimensions of sleep that are important for a wide range of clinical and research applications. Items include time the individual tried to go to sleep, duration to fall asleep, total duration of awakenings, time of final awakening and sleep quality rating. Additional items included on the diary assess whether partners shared a bed (yes/no) and after initiation of treatment, the sleep diary also includes items to assess subjective PAP use and the degree to which the couple worked together to use CPAP (rated on a scale 1–5).

##### 2.12.2.4. Sleep disturbance and sleep-related impairment.

Participants complete the PROMIS sleep disturbance and sleep-related impairment short form versions 8a [45]. Scores are presented as t-scores, with average of 50 and SD of 10. Scores >60 are considered elevated. Self-reported sleep is measured using a standardized sleep diary and the PROMIS sleep disturbance questionnaire. PROMIS items are also available with a Spanish translation.

##### 2.12.2.5. Relationship measures.

Participants complete the Couples Satisfaction Index (CSI-4) [46] to evaluate an individual's self-reported degree of satisfaction, happiness, warmth and comfort, and reward with the relationship. The 4-items on the scale are summed and range from 0 to 21. Higher scores indicate higher levels of relationship satisfaction. Participants also complete 4 items from the Quality of Relationships Inventory conflict scale to assess ratings of conflict, including anger, frequency and intensity of conflict [47]. Dyadic coping is measured by the Dyadic Coping Inventory. This 10-item measure assesses responses of the couple during times of stress [48,49].

##### 2.12.2.6. Cognitive function.

Participants complete a battery of cognitive tests to assess for specific domains of functioning at baseline and 3 months. The tests were selected to be administered within 40 min and to be able to be administered over the phone in situations where the couple could not connect to the videoconferencing platform (Zoom). Tests have validated English and Spanish versions and include: the Hopkins Verbal Learning Test (memory) [50,51], Digit span, letter-number sequencing (working memory) [52–54], semantic fluency and letter fluency (executive functioning) [55,56]. Participants also complete the cognitive failures questionnaire version 2.0 to assess for subjective cognitive problems (e.g., forgetfulness) [57,58].

##### 2.12.2.7. Additional measures administered include.

CPAP self-efficacy, which assesses participants’ belief they could persist with CPAP treatment during challenges, such as “if it made my nose stuffy”. This 9-item questionnaire asks participants to rate how likely they would be to use CPAP, from 1 to 5, in each of the situations presented [59]. This measure is also available in Spanish [60].

CPAP Tactics assess couples attempts to influence CPAP use, based on social control theory. This measure was adapted for CPAP use and assesses, positive, negative, one-sided and collaborative tactics [61].

The Epworth Sleepiness scale is a widely used measure of subjective sleepiness. In this measure, participnats report on a scale of 0–3, how likely they would be to fall asleep in different situations (e.g., lying down in the afternoon, as a passenger in a car) [62]. Scores >10 are considered to be elevated. A Spanish translation is also available for this measure [63].

The Functional Outcomes of Sleep Questionnaire-10 item is administered to assess sleep-related quality of life. Participants rate each item from 1 to 4 on how much interference they are having due to their sleep [64]. This measure is available with a validated Spanish translation [65].

The Patient Health Questionnaire-8 item (PHQ-8) [66] and the Generalized Anxiety Disorder-2 item (GAD-2) [67] measures are administered to assess for depression and anxiety symptoms. A PHQ-8 score of 10 or higher may indicate elevated depressive symptoms and a GAD-2 score of ≥3 may indicated elevated anxiety symptoms. Validated Spanish translations are available for these measures [68–70].

##### 2.12.2.8. Demographics, baseline measures and covariates.

Participants complete a brief demographic measure, including age, sex, race, ethnicity, marital status, length of relationship, country of origin, education level, employment status, household income level, number supported by that income, tobacco and alcohol use, and nights of bed-sharing per week. A brief measure is used to assess social determinants of health, such as food and housing insecuirty [71,72]. OSA severity (Apnea Hypnea Index) is extracted from medical records. Participants also complete a measure of their pre-treatment expetations for improvement with CPAP, available in English [59] and Spanish [60] and caregiver burden [73]. To assess culture-related factors, participants complete the Brief Acculturation Scale [74], the Mexican American Cultural Values Scale (familism subscale) [75], and the Hispanic Stress Inventory [25] (US or Immigrant versions), which all have English and Spanish versions.

### 2.13. Statistical analysis

Statistical analyses will be conducted using Stata v15 or higher using intent-to-treat methods. Following enrollment of the full sample, treatment group differences in baseline demographic and sleep related functioning variables will be tested and any variables that emerge as being significantly different between the two treatment groups will be tested as covariates in sensitivity analyses. We will evaluate baseline differences between study sites and consider adding study site in the statistical models. Significance tests of study hypotheses will be performed using two-sided tests with α = 0.05. Statistical analysis of study aims will be addressed using a series of multilevel models (MLMs) with repeated observations nested within individuals nested within dyads and modifications for the distributional properties of each outcome (i.e., binomial distributions for categorical adherence measures, Gaussian distributions for continuous outcomes, etc.).

#### Sample size justification:

Study sample size was primarily determined by power analyses of main adherence outcomes. Adherence outcomes (% nights with CPAP used≥4 h and numbers of hours of CPAP use per night) are available only for the patient and tests of study hypotheses depend on the precision of estimates of group effects at the highest level of nesting (i.e., the individual). As such, power estimates for tests of treatment effects on these variables were generated for standard linear models rather than MLMs. Power estimates were generated using descriptive statistics for behavioral and cognitive behavioral CPAP interventions vs. IC reported in Wozniak et al. [76] as well as data from our pilot trial of WePAP and data from a recent meta-analysis of PAP adherence in behavioral treatment trials [77]. For % nights with CPAP usage≥4 h, Nuestro Sueño adherence was assumed to be 76% and IC adherence was assumed to be 41% resulting in an assumed between-group difference of *d* = .73. Power estimates based on these assumptions indicate that a sample size of 40 couples per group will provide power of .8 or higher to detect between group differences as small as *d* = .63, indicating adequate power to detect our anticipated group differences in adherence outcomes. This power calculation is based on utilizing a sample of 40 participants and using imputation for missing data. If dropout is greater than expected (>10%), we will increase the sample size to achieve at least 90% of our planned sample. These required effect sizes are also consistent with the effect size in similar behavioral trials of CPAP [76].

#### Missing data:

Missing data mechanisms will be explored using Little's (1988) test for Missing Completely at Random [78]. We will make appropriate adjustments to models depending on whether missingness is determined to be consistent with ignorable or non-ignorable mechanisms including using Full Information Maximum Likelihood estimation methods and whether adding covariates to models would be result in a model with a conditionally-dependent dropout missingness mechanism [79,80]. We will also consider multiple imputation methods for MLMs (e.g., Bayesian Linear Imputation) for ignorable missingness mechanisms [81].

## 3. Discussion

The Nuestro Sueño program is a novel intervention, adapted to focus on Latino/a patients who are newly diagnosed with OSA and beginning PAP treatment and their partners. This program addresses a major gap in care for patients with OSA, in that no current programs are culturally adapted for Hispanic couples. Overall, few studies have focused on OSA and PAP adherence among Latinos, a population that experiences increased rates of OSA and barriers to OSA diagnosis and treatment. The integration of CHWs and a CAB from the initial stages of development of the intervention is novel and has potential to reduce health disparities in this population by ensuring the intervention content and intervention deliverers are culturally aligned with the target population [30,82]. CHWs have rarely been utilized in sleep interventions and in our study, the ability of CHWs to deliver the intervention within patients’ own language and culture represents a key adaptation of our intervention. Among minoritized patient populations that experience higher rates of medical mistrust [83], engaging a trusted community member has the potential to overcome barriers to engaging in care, including providing a bridge between patients and providers, reducing mistrust, providing and navigating financial and logistical challenges to treatment [82].

An important consideration of our study is that our patient population is diverse in a multitude of ways, including country of origin, years in the US, socioeconomic status, age and gender [84]. In addition, our intervention only focuses on patients after they have reached the sleep clinic. Further research is needed to increase screening and referral for Latino/a patients. It is estimated that approximately 93% of Hispanic women and 82% of men with OSA are not treated [1], likely highly related to language, healthcare access issues, and socioeconomic barriers [85].

The dyadic focus, including efforts to promote collaboration and support to promote PAP use in the OSA patient, as well as behavioral sleep health strategies to promote sleep health is another novel aspect of this intervention, as the vast majority of sleep interventions have been focused exclusively on the individual. Of the few exceptions that have incorporated the bedpartner [86], they have not included interventions specifically aimed at improving the partner's sleep. Determining treatment goals for both partners can benefit relationship dynamics, rather than positioning one partner purely as the “patient.” Moreover, the dyadic focus is especially relevant for Hispanic communities, where the cultural value of familismo underscores the importance of family involvement in health interventions [25]. Previous research has shown that although partner engagement is important to adherence, the effects of involvement are dependent on the quality of relationship, in that among couples with poorer relationship quality, partner engagement is less helpful [87]. Therefore, focusing on the couple's communication, such as strategies for showing support and for managing disagreements, has potential to benefit adherence as well as the relationship. In summary, this randomized pilot trial will test the feasibility of a novel intervention to reduce health disparities. Results will lead to strengthening this intervention for broader implementation and may lead to further efforts to adapt couples-based PAP interventions for other populations. Nuestro Sueño holds promise for engaging patients and partners in treatment, thus improving sleep, relationship quality, cognitive function and safety for Hispanic patients, their families and communities. Opportunities for future research in this area include: examining the intervention in a larger, fully powered sample, examining important patient subgroups (e.g., effects of gender, language), long-term adherence and more conducting more detailed analyses such as actor-partner effects to examine the key individual and dyadic processes.

## Figures and Tables

**Table 1:** Assessments and Time points.

Construct	Measure	Timepoint		
		Baseline	1 mon	3 mon

Primary outcomes				
Feasibility	completion of 3 intervention sessions		x	
Acceptability	Treatment satisfaction ratings		x	
Secondary outcomes				
PAP adherence	% of days with ≥4 h of PAP use		x	x
Sleep	Sleep duration and sleep efficiency (Fitbit)	x	x	x
	Sleep diary	x	x	x
	PROMIS sleep disturbance and sleep related impairment	x	x	x
Relationship quality				
Relationship quality	Couples' satisfaction index	x	x	x
Conflict	Quality of Relationships Inventory-conflict	x	x	x
Dyadic coping	Dyadic coping inventory	x	x	x
Other relevant outcomes				
Cognitive function	Testing battery: Hopkins Verbal Learning Test, digit span, letter-number sequencing, semantic fluency, letter fluency	x		x
	Cognitive failures	x	x	x
Other measures	Functional outcomes of sleep	x	x	x
	Epworth Sleepiness Scale	x	x	x
	CPAP Self-efficacy	x	x	x
	Patient Health Questionnaire-8	x	x	x
	General Anxiety Disorder-2	x	x	x
Covariates and baseline measures	Demographics	x	x	
	Medical comorbidities	x		
	Sleep medications	x		
	Smoking and alcohol use	x		
	SINCERE social determinants screener	x		
	PROMIS social support-4 item	x		
	Acculturation	x		
	Familismo	x		
	Hispanic Stress Inventory	x		
	CPAP expectations	x		
	Caregiver Burden	x		

**Table 2:** Intervention description.

Cross-cutting themes: 1. Education 2. Dyadic coping and goal setting 3. Enhancing communication	Session 1 Introduction	• Sleep as a shared experience • What is OSA, consequences of OSA • PAP treatment and goals • Working together to overcome challenges
	Session 2 Sleep health	• Check in on home practice • Sleep health • Strategies for promoting sleep health: Sleep habits, 2 process model • Navigating different sleep schedules • Including CPAP in the sleep routine
	Session 3 Winding down as a couple	• Check in on home practice • Troubleshooting any challenges with CPAP • Adjusting the sleep schedule • Winding down together before bed • Overcoming challenges to CPAP • Program wrap up

## Abbreviations:

OSA: Obstructive Sleep Apnea
PAP: Positive Airway Pressure
IC: Information control
